# H_2_ generated by fermentation in the human gut microbiome influences metabolism and competitive fitness of gut butyrate producers

**DOI:** 10.1186/s40168-023-01565-3

**Published:** 2023-06-15

**Authors:** Austin Campbell, Kristi Gdanetz, Alexander W. Schmidt, Thomas M. Schmidt

**Affiliations:** 1 Department of Microbiology and Immunology, University of Michigan, Ann Arbor, MI 48109 USA; 2 Department of Plant, Soil, and Microbial Sciences, Michigan State University, East Lansing, MI 48824 USA; 3 Department of Ecology & Evolutionary Biology, University of Michigan, MI 48109 Ann Arbor, USA; 4 Department of Internal Medicine, Division of Infectious Diseases, University of Michigan, MI 48109 Ann Arbor, USA

**Keywords:** Gut microbiota, Fermentation, Hydrogen gas, Butyrate, Methanogen, Resistant starch

## Abstract

**Background:**

Hydrogen gas (H_2_) is a common product of carbohydrate fermentation in the human gut microbiome and its accumulation can modulate fermentation. Concentrations of colonic H_2_ vary between individuals, raising the possibility that H_2_ concentration may be an important factor differentiating individual microbiomes and their metabolites. Butyrate-producing bacteria (butyrogens) in the human gut usually produce some combination of butyrate, lactate, formate, acetate, and H_2_ in branched fermentation pathways to manage reducing power generated during the oxidation of glucose to acetate and carbon dioxide. We predicted that a high concentration of intestinal H_2_ would favor the production of butyrate, lactate, and formate by the butyrogens at the expense of acetate, H_2_, and CO_2_. Regulation of butyrate production in the human gut is of particular interest due to its role as a mediator of colonic health through anti-inflammatory and anti-carcinogenic properties.

**Results:**

For butyrogens that contained a hydrogenase, growth under a high H_2_ atmosphere or in the presence of the hydrogenase inhibitor CO stimulated production of organic fermentation products that accommodate reducing power generated during glycolysis, specifically butyrate, lactate, and formate. Also as expected, production of fermentation products in cultures of *Faecalibacterium prausnitzii* strain A2-165, which does not contain a hydrogenase, was unaffected by H_2_ or CO. In a synthetic gut microbial community, addition of the H_2_-consuming human gut methanogen *Methanobrevibacter smithii* decreased butyrate production alongside H_2_ concentration. Consistent with this observation, *M. smithii* metabolic activity in a large human cohort was associated with decreased fecal butyrate, but only during consumption of a resistant starch dietary supplement, suggesting the effect may be most prominent when H_2_ production in the gut is especially high. Addition of *M. smithii* to the synthetic communities also facilitated the growth of *E. rectale*, resulting in decreased relative competitive fitness of *F. prausnitzii.*

**Conclusions:**

H_2_ is a regulator of fermentation in the human gut microbiome. In particular, high H_2_ concentration stimulates production of the anti-inflammatory metabolite butyrate. By consuming H_2_, gut methanogenesis can decrease butyrate production. These shifts in butyrate production may also impact the competitive fitness of butyrate producers in the gut microbiome.

Video Abstract

**Supplementary Information:**

The online version contains supplementary material available at 10.1186/s40168-023-01565-3.

## Background

Hydrogen gas (H_2_) is a common product of bacterial metabolism in anoxic environments, when electron acceptors for anaerobic respiration are limited. H_2_ is commonly produced when fermentative microbes use protons as electron acceptors to dispose of reducing power, reducing them to H_2_ via hydrogenases [1–3]. Thermodynamic principles render H_2_ production less favorable when H_2_ concentrations are high, impacting the metabolism of H_2_-producing microbes [4–6]. Hydrogenase genes occur in phylogenetically diverse microbes including 71% of the reference genomes in the Human Microbiome Project Gastrointestinal Tract database, suggesting that H_2_ concentration may be a major factor influencing fermentation in the human gut microbiota [7].

H_2_ produced during bacterial fermentation in the large intestine can be consumed by other microbes, escape in flatus, or diffuse into the blood stream where it is subsequently released into the lungs and exhaled. The summation of these processes results in a concentration of H_2_ in intestinal gas ranging from undetectable to over 40% *v*/*v* (Supplementary Fig. 1) [8, 9]. Diet is a major determinant of H_2_ production in the human colon. In particular, fermentable, microbiota-accessible carbohydrates (MACs) [10] largely drive H_2_ production [11, 12]. Despite the ubiquity of H_2_ in the environment of the large intestine, concrete information is lacking about how the concentration of hydrogen regulates fermentation of specific gut microbes. An effect of H_2_ concentration on human gut butyrogens has been predicted [13] and would be of particular significance because of the anti-inflammatory and anticarcinogenic effects of butyrate [14–17].

**Fig. 1:**
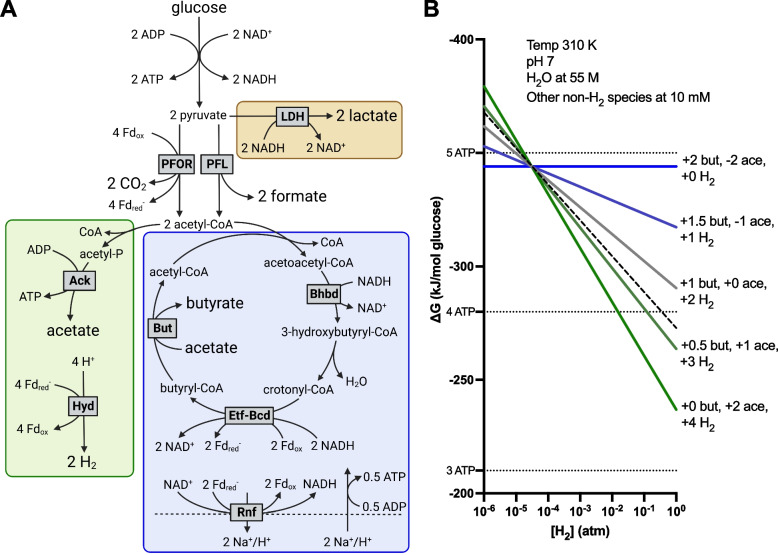
Stoichiometry and thermodynamics of fermentation in human gut butyrogens.** A** Generic fermentation pathways in human gut butyrogens that can yield combinations of H_2_, CO_2_, formate, lactate, acetate, and butyrate (adapted from Louis and Flint, 2017 [18]). Fd ferredoxin, LDH lactate dehydrogenase, PFOR pyruvate:ferredoxin oxidoreductase, PFL pyruvate formate-lyase, Ack acetate kinase, Hyd ferredoxin hydrogenase, Bhbd hydroxybutyryl-CoA dehydrogenase, Etf-Bcd butyryl-CoA dehydrogenase electron-transferring flavoprotein complex, But butyryl-CoA:acetyl CoA transferase, Rnf complex. The division of acetyl-CoA between acetate production (green box) and butyrate production (blue box) is variable; an equal division is shown as a representative case with simple stoichiometry. The division of pyruvate between the PFOR and PFL routes of acetyl-CoA formation is also variable. Stoichiometry is balanced from glucose to either lactate (orange box) or to butyrate and acetate, via either PFOR or PFL. **B** Illustration of the effect of [H_2_] on ∆G of glucose fermentation to butyrate and acetate. A range of possible fermentation balances are shown, with the net molar production or consumption of butyrate (but), acetate (ace), and H_2_ per glucose indicated. Dotted horizontal lines indicate theoretical ∆G thresholds for formation of a given number of ATP, assuming ∆G of ADP phosphorylation as + 70 kJ/mol [19]. Dashed line indicates the least butyrate formation likely to be feasible for human gut butyrogens. Representative physiological conditions were used as indicated. See Supplementary Table 4 for complete reactions and thermochemical parameters

The fermentation scheme of typical human gut butyrogens is depicted in Fig. 1A. Carbohydrate substrates (most simply represented by glucose) are first processed via glycolysis. Per glucose, the reactions of glycolysis form two pyruvate and phosphorylate two ADP to form ATP by substrate-level phosphorylation (SLP). Importantly, glucose oxidation to pyruvate reduces two moles of the cofactor NAD^+^ to NADH [18]. The necessity of regenerating NAD^+^ from this NADH to maintain redox balance represents both a central constraint on possible fermentations and an opportunity to conserve additional energy [2, 19].

Following glycolysis, pyruvate may be reduced to lactate by lactate dehydrogenase (LDH). This pathway fully reoxidizes the NADH produced in glycolysis, but produces no additional ATP [20]. More commonly, pyruvate is converted into acetyl-CoA and either CO_2_ or formate. Production of acetyl-CoA and CO_2_ is catalyzed by pyruvate:ferredoxin oxidoreductase (PFOR) and is coupled with the reduction of ferredoxin (Fd), a small iron–sulfur protein with a lower reduction potential than NAD^+^ [19, 21, 22]. By contrast, pyruvate cleavage into acetyl-CoA and formate is catalyzed by pyruvate formate-lyase (PFL) and does not generate any additional reduced species [21, 23]. Formate may subsequently be used in anabolic pathways or simply secreted as a fermentation product [23]. In vivo, both the PFOR and PFL pathways of acetyl-CoA formation can be active simultaneously [24].

As with pyruvate, acetyl-CoA in human gut butyrogens can proceed down either of two branched pathways culminating in acetate or butyrate production [2, 13, 18]. In acetate production, the acetyl group is transferred from CoA to phosphate to form acetyl phosphate (acetyl-P). In a reaction catalyzed by acetate kinase (Ack), this phosphate is then transferred to ADP to generate ATP via SLP, releasing acetate. This pathway conserves energy as ATP but does not contribute to reoxidation of NADH or reduced ferredoxin (Fd_red_^−^) [2, 13, 25]. Butyrate production, by contrast, is an important sink for reducing power. In this pathway, two acetyl-CoA are combined to form acetoacetyl-CoA, which is then reduced to 3-hydroxybutyryl-CoA by hydroxybutyryl-CoA dehydrogenase (Bhbd) in a reaction that reoxidizes one NADH cofactor to NAD^+^ [26]. The next reaction forms crontonyl-CoA, which is then further reduced to butyryl-CoA by the butyryl-CoA dehydrogenase electron-transferring flavoprotein complex (Bcd-Etf). This electron-bifurcating complex couples crotonyl-CoA reduction to the endergonic reduction of Fd_ox_ by NADH in an overall thermodynamically feasible reaction [19, 27]. The resulting Fd_red_^−^, in addition to that formed by PFOR, is reoxidized by ferredoxin hydrogenase through the reduction of protons to H_2_ [18, 28]. Sufficient flux through the butyrate production pathway results in an overabundance of Fd_red_^−^ and a shortfall of NADH. Butyrogens can conserve additional energy in this case through anaerobic respiration using the Rnf complex, which couples Fd_red_^−^ oxidation and NAD^+^ reduction (ferredoxin:NAD^+^ oxidoreductase) to cation transport across the membrane, synthesizing ATP by a chemiosmotic mechanism [19, 29]. Final release of butyrate is by exchange with free acetate catalyzed by butyryl-CoA:acetyl CoA transferase (But), which results in net acetate consumption when more acetyl-CoA is flows to butyrate production than to acetate production [18, 25, 30].

A salient feature of the branched metabolism of gut butyrogens described above is that increasing butyrate production reduces the reductant available for H_2_ formation. Conversely, increasing acetate production necessarily entails the formation of more H_2_ in order to regenerate NAD^+^ [2, 18]. As a result, thermodynamic equilibrium increasingly favors butyrate over acetate production as ambient H_2_ concentrations rise (illustrated in Fig. 1B) [6, 13, 31]. This has led to the expectation that higher gut H_2_ concentration favors butyrate over acetate production [6, 13]. While this result or similar results demonstrating the same principle have been observed in species related to human gut butyrogens [32–34], to the best of our knowledge, the effect of ambient H_2_ concentration on fermentation by the predominant human gut butyrogens *Roseburia intestinalis*, *Eubacterium rectale*, and *Faecalibacterium prausnitzii* [13, 35] has not been directly investigated, with the exception of one study that found no effect of autogenous H_2_ on cultures of *R. intestinalis* [36].

H_2_ concentration in bacterial culture can be reduced by stirring [34, 37], sparging [32], or co-culture with hydrogen-consuming microbes (hydrogenotrophs) [4, 33, 38, 39]. Under these circumstances, hydrogenases can generate more H_2_ and oxidized end products (e.g., acetate), with correspondingly less production of reduced organic end products (e.g., ethanol, butyrate) [32–34, 37, 39]. Three guilds of hydrogenotrophs colonize the human gut: methanogens, sulfate reducers, and reductive acetogens [40–44]. These hydrogenotrophs actively consume H_2_ and may therefore play an important role in regulating the H_2_ concentrations to which human gut butyrogens are exposed.

In this study, we investigate the effect of H_2_ concentration on the profile of fermentation end products of *R. intestinalis*, *E. rectale*, and *F. prausnitzii*. We find evidence that physiologically relevant variations in H_2_ concentration influence the favored routes of reductant disposal in H_2_-producing human gut butyrogens, resulting in shifts in the production of fermentation products. Specifically, exposure to high H_2_ concentrations increases production of butyrate, lactate, and formate at the expense of acetate and, presumably, CO_2_. These metabolic shifts appear to impact the competitive fitness of certain butyrogens. We propose a model where the profile of fermentation products from these taxa, and metabolically similar fermenters in the human colon, is modulated by local colonic H_2_ concentration. This, in turn, is a balance of production by fermenters and elimination by hydrogenotrophs. Finally, we report observations from a large human cohort consuming resistant potato starch as a MAC expected to stimulate fermentation and H_2_ production. Consistent with our model, we found that hydrogenotrophic gut methanogenesis was associated with decreased fecal butyrate during supplement consumption.

## Methods

### Human cohort

Results from a portion of this study’s human cohort were previously reported by Baxter et al. (2019) [45]. Participants were recruited through Authentic Research Sections of the University of Michigan BIO173 introductory biology course. Subjects were excluded based on self-reported inflammatory bowel syndrome, inflammatory bowel disease, colorectal cancer, and consumption of antibiotics in the past 6 months. De-identified human subject metadata including age and sex is provided in Supplementary Table 1.

### Microbial strains and culture

*Methanobrevibacter smithii* F1 (DSM 2374), *Faecalibacterium prausnitizii* A2-165 (DSM 17,677), and *Roseburia intestinalis* L1-82 (DSM 14,610) were obtained from the German Collection of Microorganisms and Cell Cultures GmbH (DSMZ). *Ruminococcus bromii* VPI 6883 (ATCC 27,255) was obtained from the American Type Culture Collection (ATCC). *Eubacterium rectale* A1-86 (DSM 17,629), *Bacteroides thetaiotaomicron* VPI 5482 (DSM 2079), *Bacteroides vulgatus* Eggerth and Gagnon (ATCC 8482), and *Prevotella copri* CB7 (DSM 18,205) were obtained from collaborators.

Also included in the synthetic community were the strains *Bifidobacterium adolescentis* 269–1 and *Anaerostipes caccae* 127–8-5, which are isolates from fecal samples obtained in the course of the human cohort study. *B. adolescentis* 269–1 was obtained from a fecal sample serially diluted and plated on Bifidus Selective Medium agar (BSM Agar, Sigma-Aldrich) including the BSM supplement according to the manufacturer’s instructions. Plates were incubated at 37 °C in an anoxic atmosphere of 5% carbon dioxide, between 1.5 and 3.5% H_2_, and balance N_2_ in an anaerobic chamber (Coy Laboratory Products Inc., Grass Lake, MI). *Bifidobacterium* colonies were identified as having a pink center and light brown edge and were restreaked on BSM agar.

*A. caccae* 127–8-5 was obtained from a fecal sample stored at − 80 °C in an OMNIgene-Gut collection kit tube (DNA Genotek, Ottawa, Ontario, Cat#OMR-200). The fecal sample was serially diluted and plated on SABU agar, a medium containing 2 g/L taurocholate to stimulate spore germination (full list of medium components in Supplementary Table 2). Plates were incubated at 37 °C in the anaerobic chamber described above, and colonies that grew were picked.

The taxonomic identity of the 269–1 and 127–8-5 isolates was determined using Sanger sequencing of the 16S rRNA gene. 16S rRNA was amplified using primers designated 8F (5′-AGAGTTTGATCCTGGCTCAG-3′) and 1492R (5′- GGTTACCTTGTTACGACTT-3′) and sequenced from the 8F primer. These sequencing results have been deposited in Zenodo (https://doi.org/10.5281/zenodo.6643453).

All microbial strains used in this study are available upon request made to the lead contact.

All microbial strains were preserved in frozen stocks at − 80 °C with either 5% DMSO or 20% glycerol as a cryopreservative. To begin cultivation, for all strains except *M. smithii* F1, a small amount of material was scraped from the frozen stocks and added to 5–10 mL of SAB4 base medium (components in Supplementary Table 2) supplemented with either 4 g/L D-glucose or 2 g/L each of D-glucose and D-fructose in the Coy anaerobic chamber described above. These cultures were incubated at 37 °C and passaged as necessary (no more than four passages, most commonly one or two) to produce mid- or late-exponential phase cultures used to inoculate experimental cultures. For *M. smithii* F1, frozen stocks were thawed in the anaerobic chamber and transferred using a 1-mL syringe fitted with a 23-gauge needle into a Balch tube sealed with a butyl rubber stopper and aluminum crimp (Chemglass Life Sciences, Vineland, NJ, Cat#CLS-4209) containing 5 mL SAB4 base medium under a headspace of 80% H_2_ + 20% CO_2_ mixed gas at 20 psig. These primary cultures were incubated at 37 °C on an orbital shaker at 150 rpm and passaged anaerobically as necessary to produce mid- or late-exponential phase cultures used to inoculate experimental cultures.

### Monoculture experiments

Cultures were grown at 37 °C in 10 mL (H_2_ headspace experiments) or 5 mL (CO headspace experiments) SAB4 base medium (Supplementary Table 2) supplemented with 4 g/L D-glucose and 2.31 g sodium bicarbonate in Balch tubes sealed with butyl rubber stoppers and aluminum crimps (as described in Experimental Model and Subject Details). For shaking cultures, the Balch tubes were placed on their side in an orbital shaker at 150 rpm.

All Balch tubes were prepared with a headspace of 80% N_2_ + 20% CO_2_ mixed gas at atmospheric pressure. In experiments involving the addition of H_2_ to the headspace, all cultures were prepared with a headspace at 3 atm gauge pressure containing the indicated partial pressure of H_2_ and the balance N_2_. Gases were added using a custom gas manifold, and a pressure gauge was used to adjust regulators to supply the correct pressure (SSI Technologies Inc., Janesville, WI, Cat#MG-30-A-9 V-R). Ultra-high purity grades of N_2_ and H_2_ were used. In experiments involving the addition of carbon monoxide (CO), 2.2 mL of either pure CO or N_2_ was added using a syringe fitted with a stopcock and needle. All gases used in this study were purchased from Cryogenic Gases Inc., a division of Metro Welding Supply Corp. (Detroit, MI).

Growth curves were obtained by making regular measurements of the OD_600_ in the culture tubes using a Spec-20 spectrophotometer (Thermo Spectronic Model 333,183). Before each series of measurements, the spectrophotometer was zeroed using a Balch tube containing uninoculated medium from the same batch used in the experiment.

Monoculture experiments under H_2_ were performed twice for *E. rectale* and *F. prausnitzii* and three times for *R. intestinalis*. One experiment each for *E. rectale* and *R. intestinalis* included conditions with ppH_2_ of 2 and 3 atm in addition to 0 and 1 atm. All conditions in all experiments under H_2_ had three to five biological replicates. Monoculture experiments with CO were performed once for each butyrogen with four (*R. intestinalis*, *F. prausnitzii*) or five (*E. rectale*) biological replicates.

### Synthetic community experiments

Cultures of synthetic community members (excluding *M. smithii* F1) were grown from stock in SAB4 base medium supplemented with 2 g/L each of D-glucose and D-fructose in the anaerobic chamber described above (and passaged so as to obtain mid- or late-exponential phase cultures of all the microbes simultaneously (as described above in Experimental Model and Subject Details)). Once this was achieved, equal cell numbers of each synthetic community member (estimated using OD_600_ measurements) were combined to create an inoculation mix, which was used to inoculate Balch tubes for the experimental cultures, which were subsequently sealed. These Balch tubes contained 10 mL of the SAB4 base medium supplemented with D-glucose and D-fructose described above. Since they were inoculated and sealed in the anaerobic chamber, their initial headspace matched that of the anerobic chamber (5% carbon dioxide, between 1.5 and 3.5% H_2_, balance N_2_). Each experimental culture was grown for 24 h, then passaged at a 1:100 dilution into another Balch tube for two subsequent 24-h cultures. For shaking cultures, the Balch tubes were placed on their side in an orbital shaker at 150 rpm.

Cultures of *M. smithii* F1 were grown from stock in Balch tubes and passaged so as to obtain mid- or late-exponential phase cultures at the same time as the other synthetic community members. *M. smithii* cells were added to the appropriate experimental cultures as an inoculum separate from the inoculation mix described above. Additional inocula of *M. smithii* were added at each passage of the synthetic community from pure cultures of *M. smithii *that were maintained in Balch tubes during the course of the experiment. The number of *M. smithii* cells added in each inoculum was estimated using OD_600_ and kept consistent.

The synthetic community experiment was performed twice with five biological replicates for each condition each time. Shaking cultures were only included in the second experiment.

### Synthetic community relative abundance quantification

One milliliter samples of synthetic community cultures were centrifuged for 2 min at 11,000* g*. Genomic DNA was extracted from the pellet using a DNeasy PowerLyzer Microbial Kit (Qiagen, Cat#12,255–50) according to the manufacturer’s instructions. The V4 region of the 16S rRNA gene was amplified and sequenced on the Illumina MiSeq platform using a 2 × 250-bp paired-end kit as described in Kozich et al. (2013) [46].

The resulting 16S amplicon data was analyzed using mothur v1.39.5 [47]. The mothur script and logfile have been deposited in Zenodo (https://doi.org/10.5281/zenodo.6621661). In summary, paired-end reads were merged into contigs, screened for sequencing errors, and aligned to the SILVA v132 reference database [46]. Aligned sequences were pre-clustered at 1 difference, screened for chimeras, and classified using the SILVA v132 reference database. Sequences identified as mitochondria, chloroplasts, or eukaryotes were removed. Sequences were then clustered into 99% OTUs, which reproduced the 9 community members (plus *M. smithii*) known to be present in the cultures, and a shared file was exported. Microsoft Excel (Microsoft Corporation, Redmond, WA) was used to calculate relative abundances from the shared file, and the results were imported into GraphPad Prism 9 (GraphPad Software, San Diego, CA), where statistical analyses were carried out as described in the figure legends.

### Aqueous fermentation product quantification

Samples of 1 mL bacterial culture were centrifuged for 2 min at 11,000* g* and the supernatant passed through a 0.22-µm MultiScreen_HTS_ GV 0.22-µm filter plate (Millipore Sigma, Burlington, MA). Similar to the procedure described by Baxter et al. (2019) [45], filtrates were transferred into 100-µl inserts inside 1.5-ml screw cap vials in preparation for analysis by HPLC. Quantification of SCFAs was performed using a Shimadzu HPLC system (Shimadzu Scientific Instruments, Columbia, MD) that included an LC-10AD vp pump A, LC-10AD vp pump B, DGU-14A degasser, CBM-20A communications bus module, SIL-20AC HT autosampler, CTO-10AS vp column oven, RID-10A RID detector, and an Aminex HPX-87H column (Bio-Rad Laboratories, Hercules, CA). We used a mobile phase of 0.01 N H_2_SO_4_ at a total flow rate of 0.6 ml per min with the column oven temperature at 50 °C. The sample injection volume was 10 µl, and each sample eluted for 40 min. The concentrations were calculated using standard curves generated for each product from a cocktail of short-chain organic acid standards at concentrations of 40, 20, 10, 5, 2.5, 1, 0.5, 0.25, and 0.1 mM. These standards were run before and after each batch of samples, and standard curves were generated using averaged values. The baseline of the chromatographs was manually corrected to ensure consistency between samples and standards. Samples were analyzed in a randomized order.

### Gaseous fermentation product quantification

Gas samples were removed from the headspace of cultures using syringes fitted with stopcocks. Methane content was measured using a Shimadzu GC-2014A greenhouse gas analyzer gas chromatograph (Shimadzu Scientific Instruments, Inc., Columbia, MD) equipped with a flame ionization detector (FID) fed by ultra-high purity H_2_ and zero-grade air. Ultra-high purity N_2_ was used as the carrier gas. Sample separation was performed with a 1.0-M Hayesep T 80/100 mesh column, a 4.0-M Hayesep D 80/100 mesh column, and a 0.7-M Shimalite Q 100/180 mesh column. Before each series of measurements, accuracy was checked using a 500-ppm methane standard (Argus-Hazco, Byron Center, MI, Cat#GD40-007-A-221S).

H_2_ content was measured using a Peak Performer 1 gas chromatograph (Cat#910–105) with a reducing compound photometer (RCP) detector and post-column diluter (Peak Laboratories, Mountain View, CA) calibrated using a 10-ppm H_2_ standard (GASCO 105L-H2N-10, Cal Gas Direct Incorporate, Huntington Beach, CA). Ultra-high purity N_2_ was used as the carrier gas. When necessary, samples were diluted in room air using syringes fitted with stopcocks before measurement to reduce the H_2_ concentration below 100 ppm, which was the upper detection limit.

### Total protein quantification

Total protein content was used as an indicator of bacterial biomass; 1 mL samples of microbial cultures at endpoint were centrifuged for 2 min at 11,000* g*. The supernatant fraction was stored at − 80 °C for later analysis. The pellet was resuspended in 1.5 mL distilled H_2_O and sonicated to lyse cells. Sonication was performed on ice using a Branson Digital Sonifier 450 equipped with a 102C converter and microtip, which was placed directly in the bacterial suspension; 35% amplitude was used for a 3-min cycle of 1 s on followed by 14 s off (total 12 s sonication time). Protein concentrations in the resulting lysate and the saved supernatant fraction were determined using a Pierce Coomassie Plus Bradford assay reagent (Thermo Scientific, Cat#23,238) with bovine serum albumin (BSA) standards per the manufacturer’s instructions. The results from the lysate and supernatant were added together to obtain the total protein yield of the culture.

### Human cohort study design and sample collection

The study took place during a number of separate semesters over the course of 3 years, from the winter semester of 2016 to the winter semester of 2019. While all supplements consumed consisted of resistant starch from potato (RSP), they varied in source, total dose, and frequency. The supplements consumed were Bob’s Red Mill potato starch (Bob’s Red Mill Natural Foods, Milwaukie, OR) consumed as a 20-g dose twice daily, a 20-g dose mixed with 2.5 g psyllium twice daily, a 40-g dose once daily, or a 40-g dose twice daily; or resistant potato starch from LODAAT Pharmaceuticals (Oak Brook, IL) consumed as a 20-g dose once daily. The supplement and dosage consumed by each subject is documented in Supplementary Table 1.

In each semester, the study followed a 3-week course. During the first week, fecal and breath samples were collected before consumption of RSP. During the second week, RSP consumption began at a half dose and increased to the full dose. During the third week, RSP consumption continued at the full dose while fecal and breath samples were collected.

### Human sample analysis

Fecal sample collection, preparation, and quantification of short-chain fatty acid concentration by high-performance liquid chromatography (HPLC) was performed as previously described in Baxter et al. (2019) [45]. Breath samples consisted of 30 mL of end-expiratory breath collected in a 30-mL gastight syringe. Immediately after collection, samples were injected into a QuinTron BreathTracker SC analyzer (QuinTron Instrument Company Inc., Milwaukee, WI, Cat#QTLNRBTGCSC) for analysis. Concentrations of H_2_, methane, and carbon dioxide gas were measured, and hydrogen and methane measurements were normalized based on an assumed nominal concentration of 3.5% carbon dioxide. The BreathTracker analyzer was calibrated daily using a standard calibration gas containing 150 ppm H_2_, 75 ppm methane, and 6% carbon dioxide (QuinTron, Cat#QT07500-G). The quantifications of fecal butyrate in each fecal sample and H_2_ and methane in each breath sample are provided in Supplementary Table 1.

### Microbial culture fermentation products

In monocultures of *R. intestinalis*, *E. rectale*, and *F. prausnitzii*, depending on the moles of butyrate formed per glucose fermented, acetate can be either a net product (< 1 mol butyrate per mol glucose) or net substrate (> 1 mol butyrate per mol glucose) of fermentation. Even when it is a net substrate, however, some acetyl-CoA still flows to acetate production and ATP formation by acetate kinase. In order to not obscure this nuance by reporting the net consumption of acetate that was observed in some cultures, results for fermentation products were expressed as the percent of total carbon consumed that was used in the formation of each product, rather than simple carbon recovery. This metric was calculated by first reasoning that since all three of these butyrogens produce butyrate by consuming acetate via the butyryl-CoA:acetate CoA enzyme, each mole of butyrate produced represented a mole of acetate (abundantly available in the SAB4 medium) consumed [30]. Therefore, a molar value of acetate that was theoretically consumed was set equivalent to moles of produced butyrate. Total fermented carbon was then calculated by adding the moles of carbon in the consumed glucose to the moles of carbon in theoretically consumed acetate. Total acetate produced was then calculated by adding the moles of theoretically consumed acetate to the change in acetate measured in the culture at endpoint versus blank medium, which varied from consumption to production depending on strain and condition. Since neither butyrate, formate, nor lactate were present in the medium or expected to be consumed during microbial metabolism, their total quantity produced was simply taken to be their endpoint concentration. Percent total fermented carbon in each substrate was then calculated as the moles of carbon in the produced substrate divided by the total moles of fermented carbon.

In the synthetic communities, the presence of diverse potential metabolic pathways rendered the above approach impractical. Instead, results for each fermentation product were expressed as change in the product in moles (an increase in all but one culture where acetate decreased) divided by the total moles of substrate (glucose and fructose) consumed in the culture.

All statistical analyses of metabolite data obtained from microbial cultures were performed using GraphPad Prism 9 and are described in the figure legends.

### Human samples and methanogenesis classification

Concentrations of fecal metabolites obtained from HPLC (described above) were normalized to the wet weight of fecal material. Concentrations of CH_4_ and H_2_ in breath samples were quantified as described above. For each fecal metabolite and breath gas, samples with values lying more than three interquartile ranges below the lower quartile or above the upper quartile were excluded from analysis according to the method of Tukey’s Fences [48].

Subjects were classified as methanogenic or non-methanogenic, with separate classifications for the periods before and during supplement consumption. Methanogenic subjects were defined as those with over 4 ppm methane in at least one breath sample. This cutoff was based on a study of responses to consumption of lactulose (a fiber inaccessible to human enzymes but rapidly degraded by the gut microbiota) which suggested that a baseline threshold of 4 ppm above background is best predictive of increased breath methane [49]. We used this threshold because our intent was to identify subjects where methanogenesis was not just present, but a significant component of the gut ecosystem. In the Winter 2016 and Winter 2019 semesters, an elevated baseline concentration of 1 ppm methane was observed across most samples. Since this was likely due to instrument calibration rather than biological activity, this elevated baseline was subtracted before classifying individuals as methanogenic or non-methanogenic. The average concentration of each fecal metabolite and breath gas before and during starch supplement consumption was then calculated for each subject. The average concentration of fecal metabolites and breath gases in methanogenic and non-methanogenic subjects was then compared using two-tailed Student’s *t*-tests in GraphPad Prism 9.

## Results

To test the hypothesis that H_2_ modulates the production of fermentation products by human gut butyrogens, we studied pure cultures of strains representing abundant butyrogens in the human gastrointestinal tract. *Eubacterium rectale* A1-86 and *Roseburia intestinalis* L1-82 were selected to represent the generalized metabolic pathways of butyrogens that could be affected by H_2_ (Fig. 1). *Faecalibacterium prausnitzii* A2-165 was chosen as a representative of butyrogens that lack a hydrogenase and are therefore unlikely to be affected by H_2_.

**Fig. 2:**
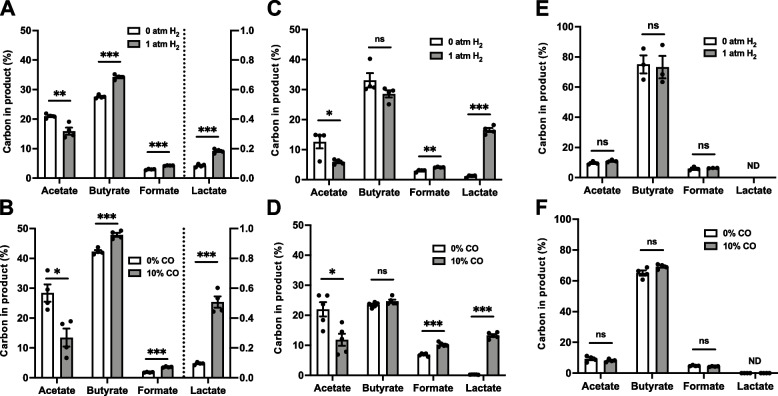
Variation in fermentation products in cultures of human gut butyrogens grown under different atmospheres. Endpoint fermentation products in cultures of *R. intestinalis* (note lactate is shown on smaller scale on right axis) (**A–B**), *E. rectale* (**C–D**), and *F.* prausnitzii (**E–F**) grown in shaken cultures with H_2_, N_2_, or CO—a potent inhibitor of hydrogenases. Error bars indicate SEM. Statistical significance calculated using two-sided Student’s two-sample *t*-tests (*p < 0.05; **p < 0.01; ***p < 0.001)

Replicate cultures of these three butyrogens were grown under a headspace of either H_2_ or N_2_ and shaken continuously to equilibrate headspace gases with the culture medium. As predicted, the presence of a H_2_ headspace shifted the profile of fermentation away from acetate towards more reduced organic acids (e.g., lactate and butyrate) for both H_2_-producing butyrogens (Fig. 2A, C). The same pattern of fermentation products was recapitulated in the presence of carbon monoxide (CO; Fig. 2B, D), a potent inhibitor of ferredoxin hydrogenase [50]. The profiles of reduced organic acids differed between the hydrogen-producing butyrogens. In cultures of *R. intestinalis*, reducing power was diverted to butyrate and formate. Lactate production increased very significantly, but remained only a trace product in both conditions (< 0.5% substrate carbon). By contrast, cultures of *E. rectale*, saw reducing power diverted primarily to lactate, with a smaller diversion to formate and no change in butyrate. Since formate production reduces intracellular Fd_red_^−^ versus the alternative production of CO_2_, it clearly represents a diversion reducing power away from H_2_ production via ferredoxin hydrogenase, as does butyrate production (Fig. 1A). Increasing the partial pressure of H_2_ in the headspace up to 3 atm led to larger shifts in a rough dose–response pattern (Supplementary Fig. 2). Unlike *E. rectale* and *R. intestinalis*, *F. prausnitzii* lacks hydrogenase activity [51]. As expected, its fermentation products were unaffected by the presence of H_2_ or CO in the headspace (Fig. 2E, F).

To assess whether H_2_ has an impact on butyrate production in more complex microbial communities, we assembled a synthetic community of microbes isolated from the human gut. This mixture consisted of representative strains of common butyrate producers (*F. prausnitzii*, *E. rectale*, *R. intestinalis*, and *Anaerostipes caccae*), two common fiber degraders (*Bifidobacterium adolescentis* and *Ruminococcus bromii*), and several members of the abundant gut phylum Bacteroidetes (*Bacteroides vulgatus*, *Bacteroides thetaiotaomicron*, and *Prevotella copri*). We compared butyrate production by this community from equimolar quantities of glucose and fructose in the presence or absence of *M. smithii*, the predominant H_2_-consuming methanogen in the human gut [52, 53].

**Fig. 3:**
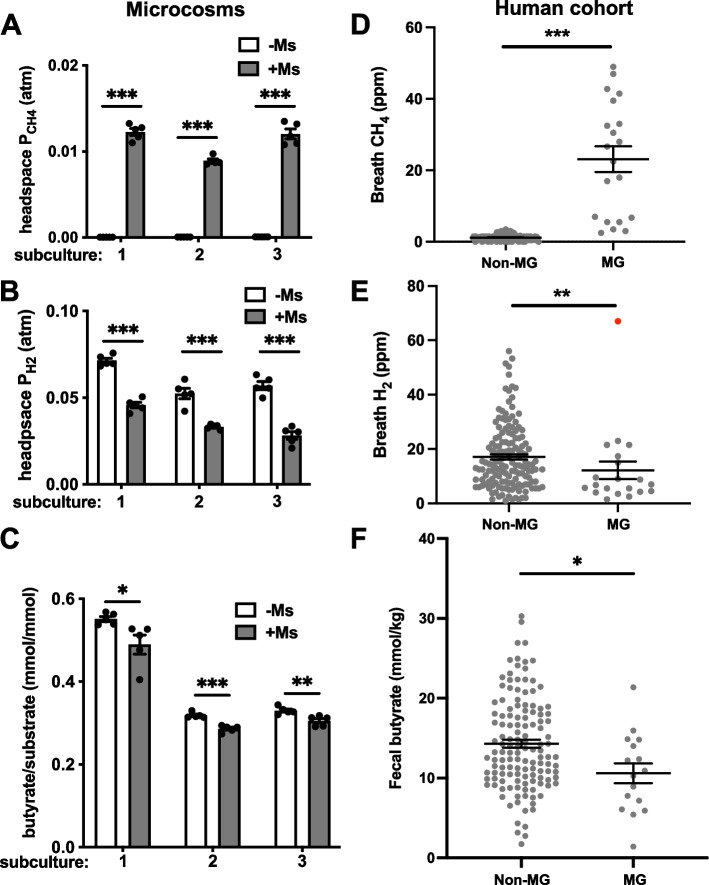
Influence of methanogenesis on butyrate production products in a synthetic gut community and the human gut microbiota. Methane (**A**), H_2_ (**B**), and butyrate (**C**) production by a 9-species synthetic human gut community grown with (shaded bars) and without (open bars) the addition of *M. smithii*. Butyrate was measured in three successive subcultures, with a new inoculum of *M. smithii* added at each passage. In a human cohort consuming a resistant potato starch supplement, breath and fecal samples were used to determine weekly average breath CH_4_ (**D**), breath H_2_ (**E**), and fecal butyrate (**F**) in individuals with and without active gut methanogenesis, defined as at least one breath methane measurement greater than 4 ppm CH_4_ in the measurement period (MG) or no breath measurements over 4 ppm in the same period (non-MG). Error bars indicate SEM. Statistical significance calculated using two-sided Student’s two-sample *t*-tests (*p < 0.05; **p < 0.01; ***p < 0.001). One breath H_2_ measurement shown in the methanogenic group with a value over 60 ppm (data point shown in red) was excluded from statistical analysis

The relative abundances of the constituent microbes, as well as production of CH_4_, H_2_, and fermentation products, were monitored over the course of three consecutive subcultures of the synthetic community. The addition of *M. smithii* resulted in the production of methane (Fig. 3A) and decreased the concentration of H_2_ and production of butyrate as predicted (Fig. 3B, C). The corresponding increase in acetate, which was observed in monocultures, was only observed in the second subculture of the synthetic community (Supplementary Fig. 3D). The expected shift in acetate production by butyrogens may have been masked by the copious production of acetate by other members of the synthetic community, such as the two *Bacteroides* species. Lactate and formate were also produced, along with the characteristic *Bacteroides* fermentation products propionate and succinate (Supplementary Fig. 3E-H). Lactate production was reduced by the addition of *M. smithii*. Since *M. smithii* consumes all available formate, its impact on formate production could not be determined.

**Fig. 4:**
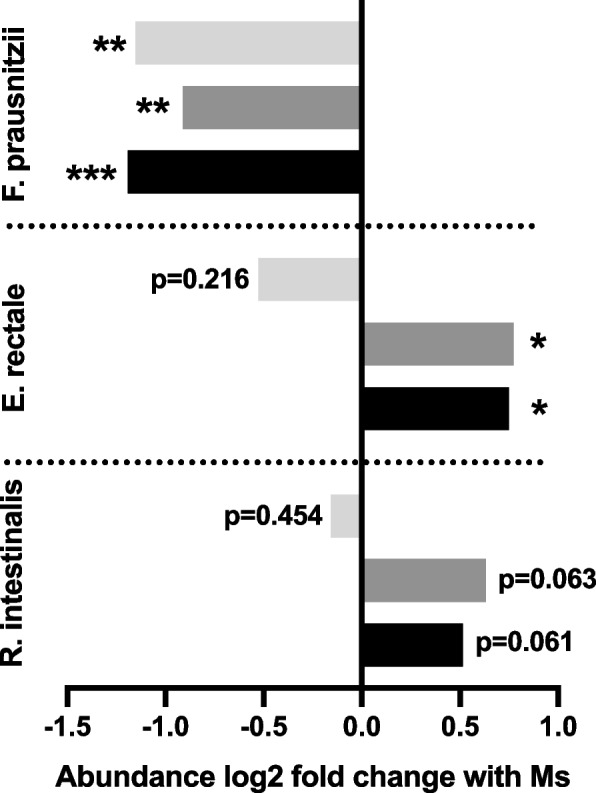
Influence of methanogenesis on competitive fitness of butyrogens in a synthetic gut community. Fold change in relative abundance of *F. prausnitzii*, *E. rectale*, and *R. intestinalis* in a 9-species synthetic human gut community with the addition of *M. smithii* compared to the same community without *M. smithii*. Relative abundance was quantified at the end of three successive 24-h subcultures (first subculture in light gray, second in dark gray, third in black), with a new inoculum of *M. smithii* added to the appropriate cultures at each passage. For each species, there were five replicate cultures in each condition. Statistical significance calculated from relative abundance values using two-sided Student’s two-sample *t*-tests (*p < 0.05; **p < 0.01; ***p < 0.001)

Removal of *M. smithii* from the synthetic community increased H_2_ levels and favored the growth of *F. prausnitzii* (Fig. 4). The increase of *F. prausnitzii* suggests that accumulation of H_2_ in the absence of a methanogen forced butyrogens with ferredoxin hydrogenase to shift their fermentation towards less energetically favorable pathways. Consistent with this explanation is the decreased abundance of *E. rectale* (Fig. 4), which had a slower growth rate and lower yield under higher H_2_ in monoculture (Supplementary Table 3). In the absence of a methanogen, *R. intestinalis* exhibited a similar, but less obvious, decrease in abundance (Fig. 4), although its growth rate and yield were not significantly impacted by high H_2_ in monoculture (Supplementary Table 3).

Incubation of the synthetic community cultures with vigorous shaking completely abrogated the effect of *M. smithii* on butyrate production and the relative abundances of butyrate producers (Supplementary Fig. 3C, I). This may be because local accumulation of dissolved H_2_ is prevented when H_2_ in the culture medium is rapidly equilibrated with the headspace, preventing H_2_ consumption by *M. smithii* from making a difference by reducing this accumulation. Shaking entirely prevented net production of lactate, which also suggests decreased exposure of the butyrogens to high H_2_ (Supplementary Fig. 3).

To explore whether the influence of *M. smithii* on butyrate production is relevant in the human gut, we collected breath samples from a human cohort both before and during consumption of resistant starch from potatoes (RSP). Resistant starch is not degraded by human amylases and reaches the gut microbiota undigested, where it can serve as a substrate for fermentation. We previously reported that RSP supplementation in a portion of this cohort increased fecal butyrate overall [45]. In this study, measurement of breath methane was used to assess gut methanogenesis both before and during RSP consumption. During RSP consumption, active gut methanogenesis was associated with lower levels of breath H_2_ (Fig. 3E) and lower fecal butyrate concentration (Fig. 3F) compared to individuals lacking gut methanogenesis. These findings are consistent with the results from in vitro cultures and suggest that the H_2_ produced from RSP breakdown in the gut may play an important role in the stimulation of butyrate production by the microbiota. Indeed, individuals with gut methanogenesis did not follow overall trend of increased fecal butyrate during RSP consumption (Supplementary Fig. 4D). Removal of H_2_ by hydrogenotrophs such as *M. smithii* appears to shift butyrogen metabolism in vivo as well as in vitro. Interestingly, gut methanogenesis in the same individuals before RSP consumption was not associated with decreased breath H_2_ or fecal butyrate (Supplementary Fig. 4A–C).

## Discussion

The in vitro results reported in this study revealed shifts in fermentation products of human gut butyrogens grown under a headspace containing 1 atm partial pressure of H_2_. While this quantity of H_2_ is not found in intestinal gas, the H_2_ concentration relevant for microbial physiology is not that of the gas above a microbial culture, but rather that of the H_2_ dissolved in the aqueous phase where microbes dwell [41, 54]. Studies in bioreactors have shown that H_2_-producing microbial communities experience dissolved H_2_ concentrations many times greater than equilibrium with the headspace, with reports indicating 3- to 100-fold overconcentrations in various conditions and bioreactor designs [54–57]. Since H_2_ in intestinal gas (analogous to bioreactor headspace gas) ranges from < 1% to > 40% (*v*/*v*) with a median of approximately 15% (Supplementary Fig. 1) [8, 9], it is likely that dissolved H_2_ in the human colon ranges above and below that produced by equilibration with 1 atm H_2_. Therefore, the metabolic shifts induced in human gut butyrogens by the 1 atm H_2_ headspace used in our in vitro cultures could also occur in vivo. Observations from a human cohort were consistent with this hypothesis (Fig. 3D–F).

Much of the H_2_ produced in the human gut is consumed in situ by hydrogenotrophic microbes [40, 41]. Accordingly, in this study, we investigated the consumption of H_2_ by gut methanogens, reasoning that active methanogenesis must at some level lead to a reduction in dissolved H_2_. The fact that nearly all CH_4_ in the human gut is produced by the single culturable species, *Methanobrevibacter smithii* [53, 58], allowed us to use a simple, in vitro synthetic gut community to model the influence of methanogenesis on fermentation in the human gut.

Previous studies of the role of gut methanogenesis have often focused on aspects of human health and sometimes produce conflicting results [44, 59]. Explaining these inconsistencies, and distinguishing between correlation and causation, is difficult without mechanistically founded expectations about the effect of H_2_ removal on the gut microbiota [59]. In the current study, we hoped to obtain more interpretable results by first studying pure cultures of important gut microbes to test theoretical expectations (Fig. 2). Establishing the effects of H_2_ concentration in this system allowed us to develop predictions for highly simplified synthetic gut communities in which H_2_ was modulated by *M. smithii* (as in the gut) rather than by direct experimental manipulation of the headspace gas (Fig. 3A–C). Finding a methanogen-mediated decrease in butyrate production in this system in turn allowed us to understand the observation of lower fecal butyrate in methanogenic individuals (previously reported by Abell et al. (2009) [60] in a small cohort of eight individuals) as consistent with the predicted effect of gut methanogens rather than simply an intriguing association (Fig. 3D–F). Notably, the increase in lactate production observed in *E. rectale* under high H_2_ likely also drives increased fecal butyrate given that lactate in the human colon appears to be rapidly fermented to SCFAs including butyrate [61–63]. Certain human gut butyrogens, notably *Anaerostipes caccae* and *Eubacterium hallii* appear to specialize in this route of butyrate production when lactate is available, while *R. intestinalis*, *E. rectale*, and *F. prausnitzii* have not been observed to significantly utilize lactate as a substrate [64, 65]. Our synthetic gut community included *A. caccae* and so provided an in vitro model of the process.

A previous study also reported decreased butyrate and increased acetate production by human gut butyrogens in the prevalent but low-abundnace genus *Christensenella* in in vitro co-culture with *M. smithii* [66]. The reported shift in fermentation was similar to our findings in *R. intestinalis*, indicating that the effects of H_2_ concentration and methanogenesis we describe are common to other human gut butyrogens beyond the strains we investigated. Another study failed to find any influence of co-culture with *M. smithii* on *R. intestinalis* fermentation and found that co-culture with the hydrogenotrophic acetogen *Blautia hydrogenotrophica* actually increased butyrate production [67]. However, these results were driven by acetate availability, as acetate was not provided in the culture medium and net acetate consumption is required for production of high levels of butyrate (Fig. 1). This likely does not reflect the environment of the human colon, where acetate is abundant [68]. The most direct evidence to date of *M. smithii* modulating fermentation in vivo does not involve a butyrogen, but rather the commonly studied *Bacteroides thetaiotaomicron*. A study using gnotobiotic mice showed that *M. smithii* modulates *B. theta* fermentation products in vivo, increasing acetate and formate production at the expense of propionate, which the authors interpreted as due to consumption of H_2_ and/or formate by *M. smithii* [69].

In the present study, simple in vitro experiments with single species allowed a more specific description of the influence of H_2_ removal on human gut fermentation beyond the commonly repeated broad description of it as facilitating, enhancing, or improving the efficiency of human gut fermentation on the whole [44, 52, 70–73]. The principle that H_2_ removal facilitates H_2_-producing fermentation in the human gut is well-founded and accounts for the decrease in competitive fitness of the hydrogenogenic butyrogens *E. rectale* and (marginally) *R. intestinalis* in the synthetic community experiments reported here (Fig. 4), as well as the impairment of *E. rectale* growth rate and yield under very high H_2_ (Supplementary Table 3). However, this perspective obscures the fact that H_2_ accumulation does not simply shut down fermentation in the human gut, as it does in other well-studied systems such as sewage digesters. There, endergonic oxidation of butyrate and propionate to acetate requires an intimate syntrophic association between fermenters and methanogens [4, 31]. Our findings show that unlike these obligate syntrophs, the predominant human gut butyrogens *E. rectale* and *R. intestinalis* [13, 35] can cope with elevated H_2_ by disposing of reducing equivalents via butyrate and lactate instead. They do suffer some loss of metabolic efficiency, especially in the case of *E. rectale* which forgoes roughly half of the its ATP formation per glucose with its dramatic shift from butyrate and acetate production to lactate fermentation. However, they continue to grow using “backup” metabolic strategies. They are therefore examples of “facultative syntrophs” [5] for whom H_2_ accumulation results in a fermentation shift rather than a fermentation arrest. Counterintuitively, high H_2_ concentration actually *stimulates* production of the fermentation products butyrate and lactate in these organisms. As predicted by estimates of the Gibbs free energy of a range of fermentation balances (Fig. 1B), exposure to increasing concentrations of H_2_ shifted fermentation products in a roughly dose–response fashion, showing that the shift is progressive and not governed by a fixed H_2_ threshold (Supplementary Fig. 2).

The reduced fecal butyrate we report in methanogenic individuals only appears during consumption of an RSP supplement (Fig. 3F) and is not observed in the same individuals before supplement consumption (Supplementary Fig. 4A–C). The most likely explanation of this result is that RSP consumption is necessary in most individuals to stimulate sufficient production of H_2_ in the colon to change the thermodynamic situation if not efficiently removed. This possibility is supported by higher average H_2_ during versus before RSP consumption (*p* = 0.005). Another explanation, not mutually exclusive with the first, is based on the biogeography of methanogens in the human colon. A number of reports indicate that methanogens are more abundant in the distal colon and rectum rather than the proximal colon [43, 74–76]. As a refractive substrate, RSP may reach the distal colon in higher quantity than most other substrates in the diet before supplementation. Therefore, RSP fermentation could be more influenced by methanogens than fermentation of substrates that are mostly degraded before reaching the distal colon. Other guilds of human gut hydrogenotrophs—the sulfate reducers and reductive acetogens—may play a greater role in modulating fermentation of these substates. Further work should seek to include these guilds of hydrogenotrophs in our understanding of the role of H_2_ in modulating fermentation in the gut microbiome.

A final point of consideration is the negative association between active gut methanogenesis and successful stimulation of butyrate by RSP supplementation. While RSP supplementation generally increased fecal butyrate [45], methanogenic individuals showed no increase in fecal butyrate on average (Supplementary Fig. 4D). Although this is a correlative finding, this study provides a theoretical basis for a causal role of methanogenesis in decreasing butyrate production via efficient H_2_ removal. Given the myriad positive effects of butyrate on colon health [14], consideration should be given to reducing methanogenesis (and perhaps hydrogenotrophy in general) during supplement interventions intended to stimulate butyrate production. An alternative approach would be to administer H_2_ to stimulate butyrate production directly. A large body of research has studied H_2_ administration for its apparent antioxidant and anticarcinogenic effects, often via the consumption of water supersaturated with H_2_ [77–79]. Our findings in this study raise the possibility that these treatments may also stimulate butyrate production in the gut microbiota, especially in combination with supplement interventions.

## Conclusions

H_2_ has often been proposed as a regulator of metabolic processes in the human gut microbiota [44], but concrete information is lacking on its specific role in the complex gut ecosystem. In this study, we examined the effect of H_2_ concentration on one prominent aspect of the human gut microbiota: production of the anti-inflammatory and anti-carcinogenic bacterial metabolite butyrate. Using in vitro approaches, we were able to observe the effect of H_2_ on three prominent human gut butyrogens: *R. intestinalis*, *E. rectale*, and *F. prausnitzii*. We found that high H_2_ concentration upregulated butyrate production by *R. intestinalis*, but not in *E. rectale*, which instead upregulated lactate production. *F. prausnitzii* was unaffected by H_2_. We further found that H_2_ consumption by the predominant gut methanogen *M. smithii* was sufficient to alter butyrate production by the H_2_-regulated butyrogens. Findings from a large human cohort supported a model in which gut H_2_ concentration, which is a balance between H_2_ production by fermenting bacteria and H_2_ consumption by methanogens, influences the total butyrate production by the gut microbiota.

## Supplementary Information

**Additional file 1:  Figures S1 -Figure S4.**

**Additional file 2:  Table S1.**

**Additional file 3:  Table S2.**

**Additional file 4:  Table S3.**

**Additional file 5:  Table S4.**

## Data Availability

16S V4 amplicon sequences generated from synthetic community cultures grown in the course of this study have been deposited in the NCBI Sequence Read Archive (SRA) as BioProject ID PRJNA956530 (https://www.ncbi.nlm.nih.gov/sra/PRJNA956530). 16S amplicon data was analyzed using mothur v1.39.5 [47]. The mothur script used and logfile generated have been deposited in Zenodo (https://doi.org/10.5281/zenodo.6621661). All other data supporting the conclusions of this study is available in the paper and supplemental materials. In particular, the de-identified human cohort data used in this study is available in Supplemental Table 2. Any additional information required to reanalyze the data reported in this paper is available from the lead contact upon request.
