# Supplementary material for: H2 generated by fermentation in the human gut microbiome influences metabolism and competitive fitness of gut butyrate producers
Source: Microbiome. 2023 Jun 15;11:133. doi: 10.1186/s40168-023-01565-3 (PMC10268494; doi:10.1186/s40168-023-01565-3)
Supplement: Supplementary file 2 — Additional file 1: Figures S1 -Figure S4. [file 40168_2023_1565_MOESM1_ESM.pdf]

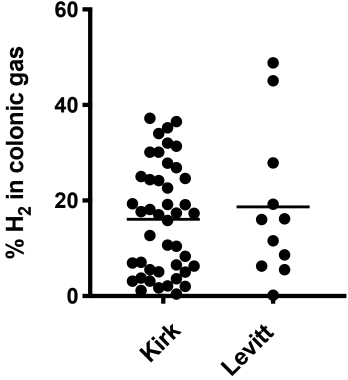

Supplementary Figure 1. H_2 measured in human intestinal gas (v/v) by two different methodologies. Kirk (1949) [8] collected total flatus produced during a 10-hour period, while Levitt (1971) [9] washed out and collected the total gas content of the large and small intestines by infusion of argon into the jejunum.

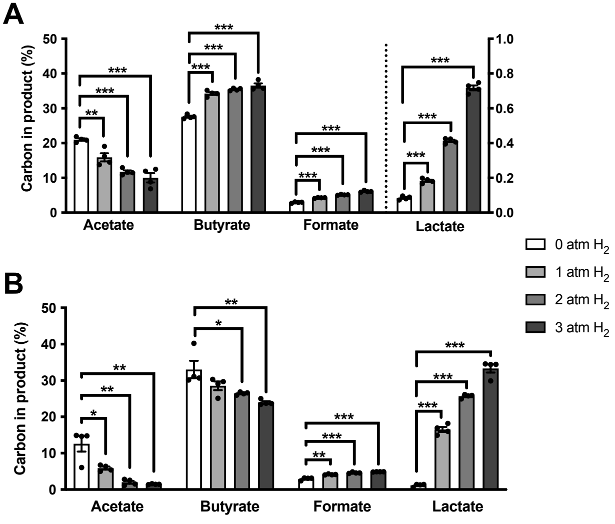

Supplementary Figure 2. Dose response of fermentation products of human gut butyrogens to headspace H_2. Endpoint fermentation products in cultures of *R. intestinalis* (A) and *E. rectale* (B), grown in shaken cultures with increasing partial pressure of H_2 in the culture headspace. Total headspace pressure was 3 atm in all conditions, with N_2 used as the balance gas. Error bars indicate SEM. Statistical significance calculated using two-sided Student's two-sample t-tests (* = $p<0.05$; ** = $p<0.01$; *** = $p<0.001$).

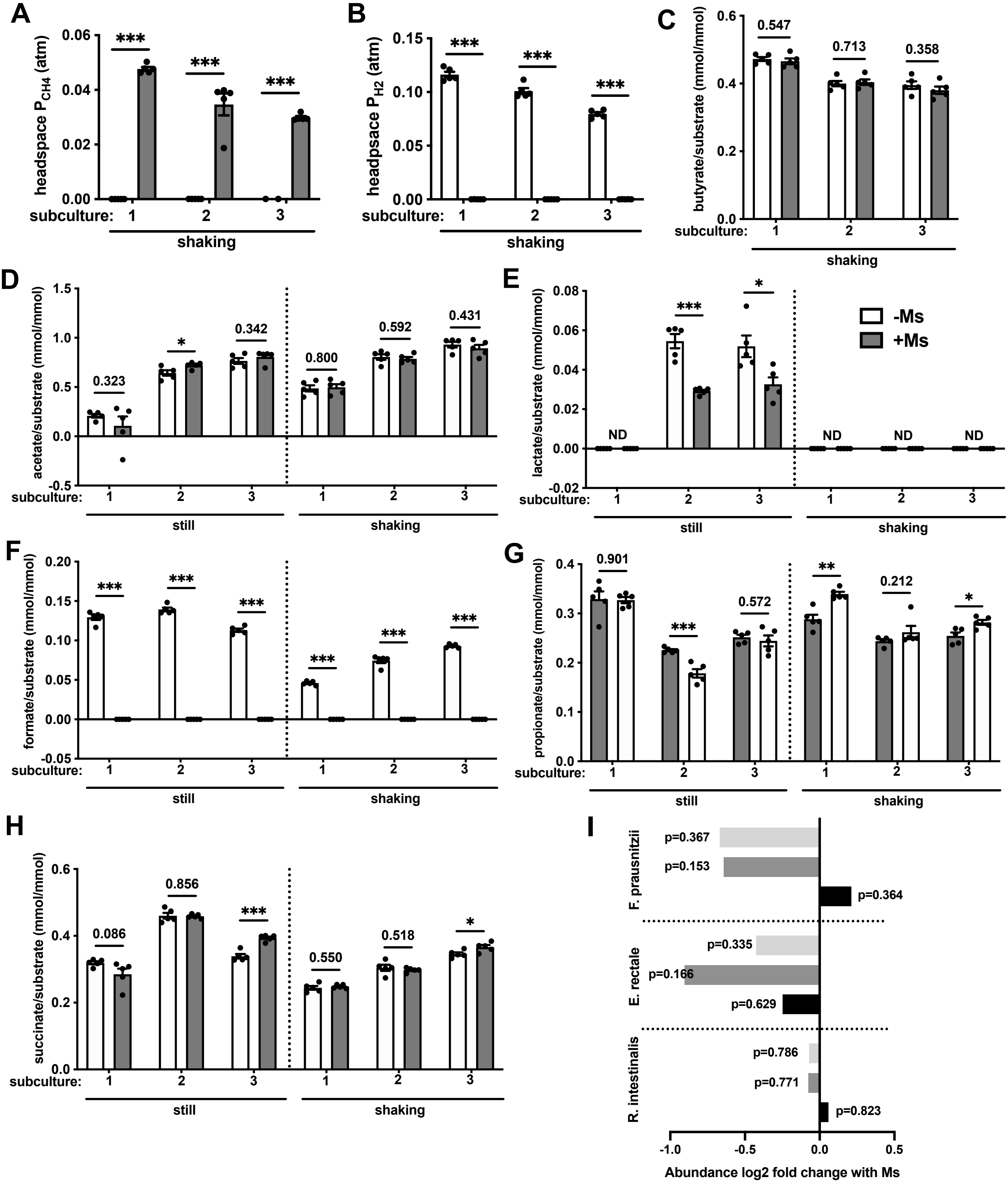

Supplementary Figure 3. Influence of methanogenesis on fermentation and growth of a synthetic gut community with variable agitation. Production of methane (A), H_2 (B), butyrate (C), acetate (D), lactate (E), formate (G), and succinate (H) by a 9-species synthetic human gut community grown with (shaded bars) and without (open bars) the addition of *M. smithii*. Cultures incubated on an orbital shaker at 150 rpm; production in still cultures is also shown for all products except butyrate. Note that formate is consumed by *M* smithii, accounting for its absence from +Ms cultures. Products were measured in three successive subcultures, with a new inoculum of *M. smithii* added at each passage. Error bars indicate SEM. Statistical significance calculated using two-sided Student's two-sample t-tests (* = p<0.05; ** = p<0.01; *** = p<0.001). Fold change in relative abundance of *F. prausnitzii*, *E. rectale*, and *R. intestinalis* in shaking synthetic community cultures (I). Statistical significance calculated from relative abundance values using two-sided Student's two-sample t-tests.

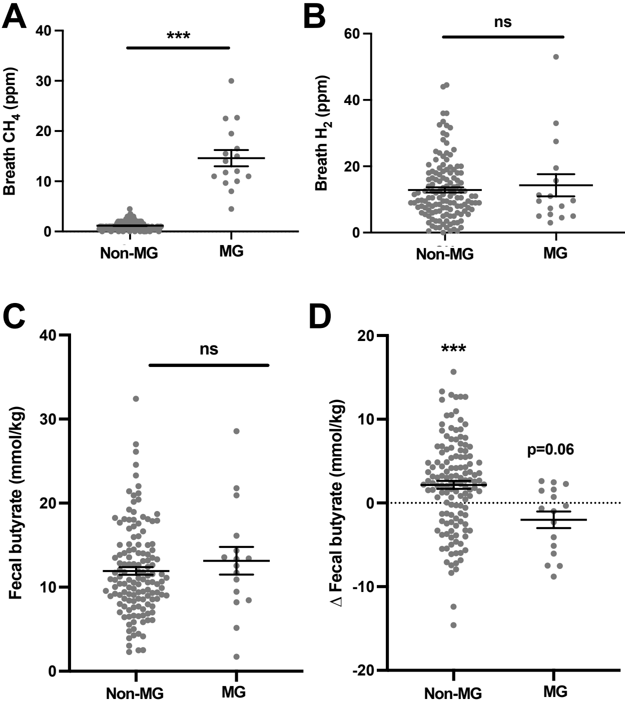

Supplementary Figure 4. Influence of methanogenesis on breath H_2 and fecal butyrate before consumption of an RSP supplement. In a human cohort before consumption of an RSP supplement, breath and fecal samples were used to determine weekly average breath CH_4 (A), breath H_2 (B), and fecal butyrate (C) in individuals with and without active gut methanogenesis. Fecal butyrate measurements taken during subsequent consumption of RSP were used to calculate the change in fecal butyrate associated with RSP supplementation (D). Error bars indicate SEM. Statistical significance calculated using two-sided Student's two-sample t-tests in A-C and one-sided t-tests for difference from a mean of zero in D (* = $p<0.05$; ** = $p<0.01$; *** = $p<0.001$).
