# Supplementary material for: H2 generated by fermentation in the human gut microbiome influences metabolism and competitive fitness of gut butyrate producers
Source: Microbiome. 2023 Jun 15;11:133. doi: 10.1186/s40168-023-01565-3 (PMC10268494; doi:10.1186/s40168-023-01565-3)
Supplement: Supplementary file 5 — Additional file 4: Table S3. [file 40168_2023_1565_MOESM4_ESM.pdf]

Supplementary Table 1		3 atm N2	3 atm H2
**Generation time (min)**	*F. prausnitzii*	237.0 (11.7)	240.2 (11.5)
	E. rectale	**66.8 (5.9)*** **	**93.9 (6.4)*** **
	R. intestinalis	72.1 (9.6)	78.8 (8.3)
**Protein biomass yield (mg)**	*F. prausnitzii*	0.544 (0.063)	0.538 (0.041)
	E. rectale	**0.225 (0.016)***	**0.204 (0.003)***
	R. intestinalis	0.247 (0.001)	0.259 (0.015)

Supplementary Table 3. Influence of H_2 on growth rate and yield of human gut butyrogens. Generation time in exponential phase and yield (quantified by total protein) of the common human gut butyrogens *F. prausnitzii*, *E. rectale*, and *R. intestinalis* grown under a headspace containing 3 atm of either N_2 or H_2. Asterisks and bold indicate a significant difference under H_2 versus N_2 (Student's t-test, $p<0.05$). Standard deviation is shown in parentheses. Four replicate cultures of *F. prausnitzii* and *E. rectale* were present in each condition; five replicate cultures of *R. intestinalis* were present in each condition.
